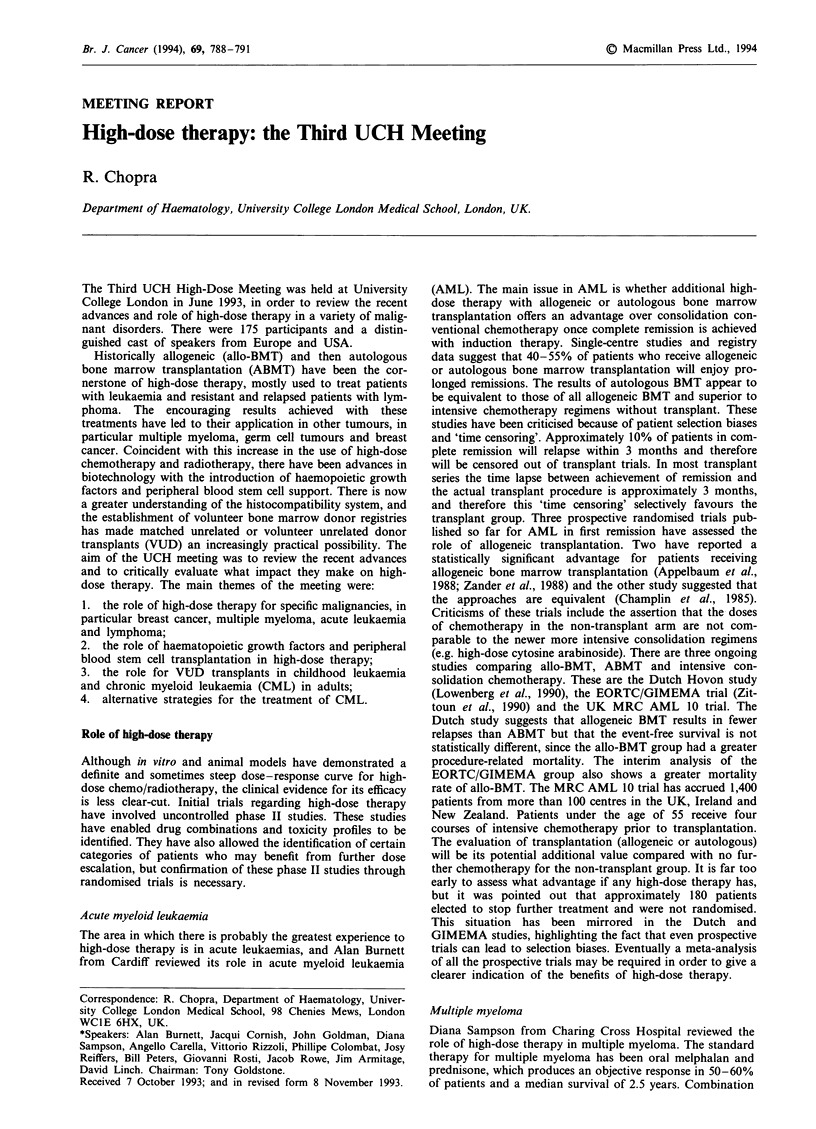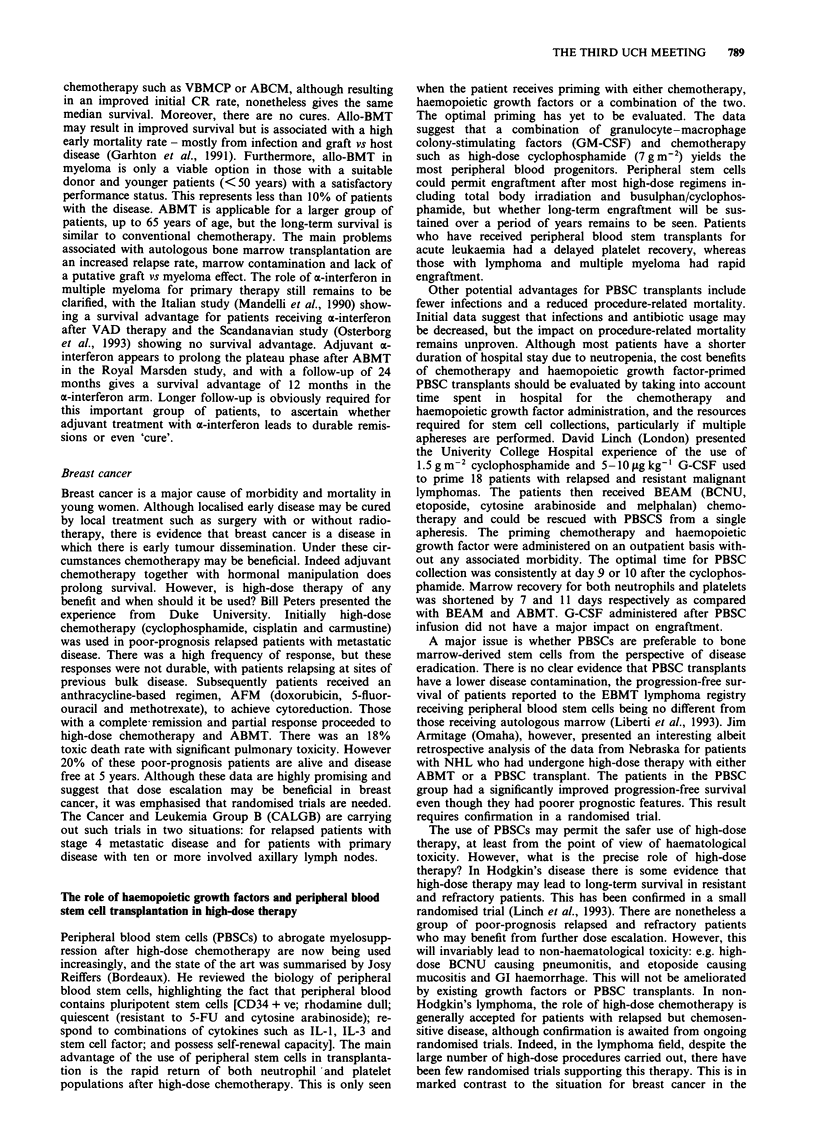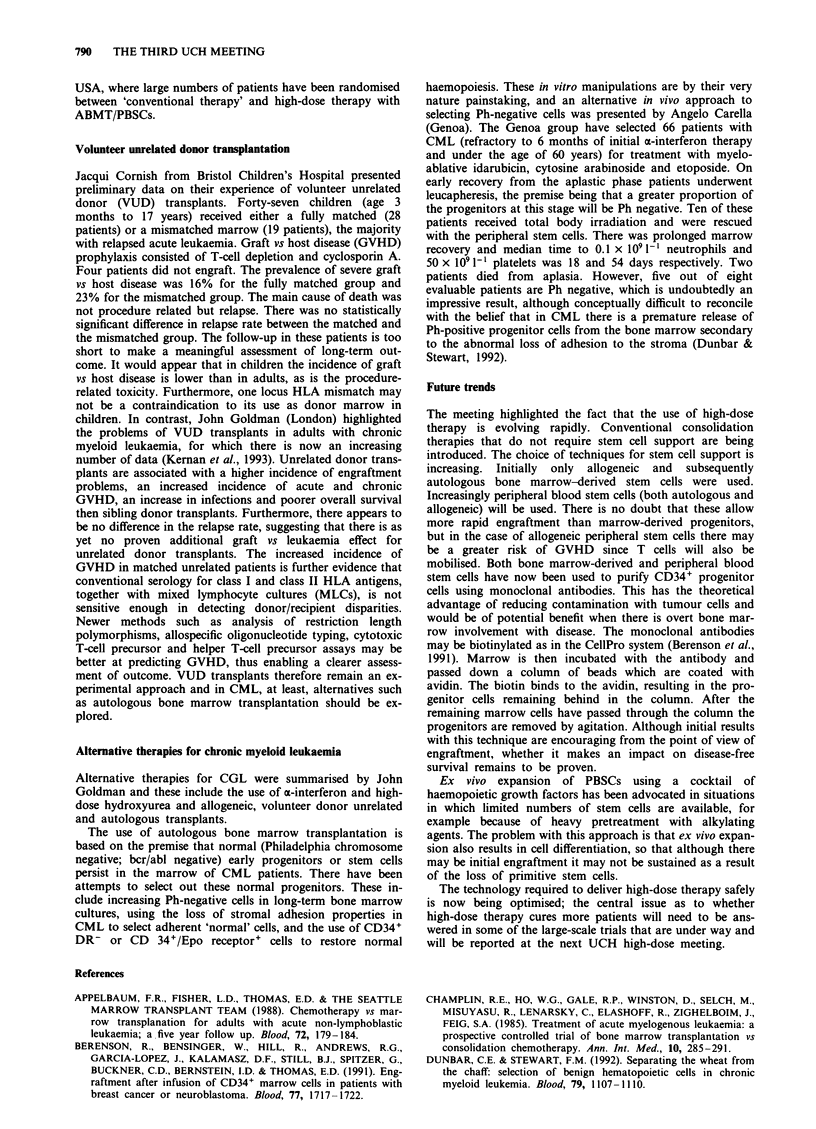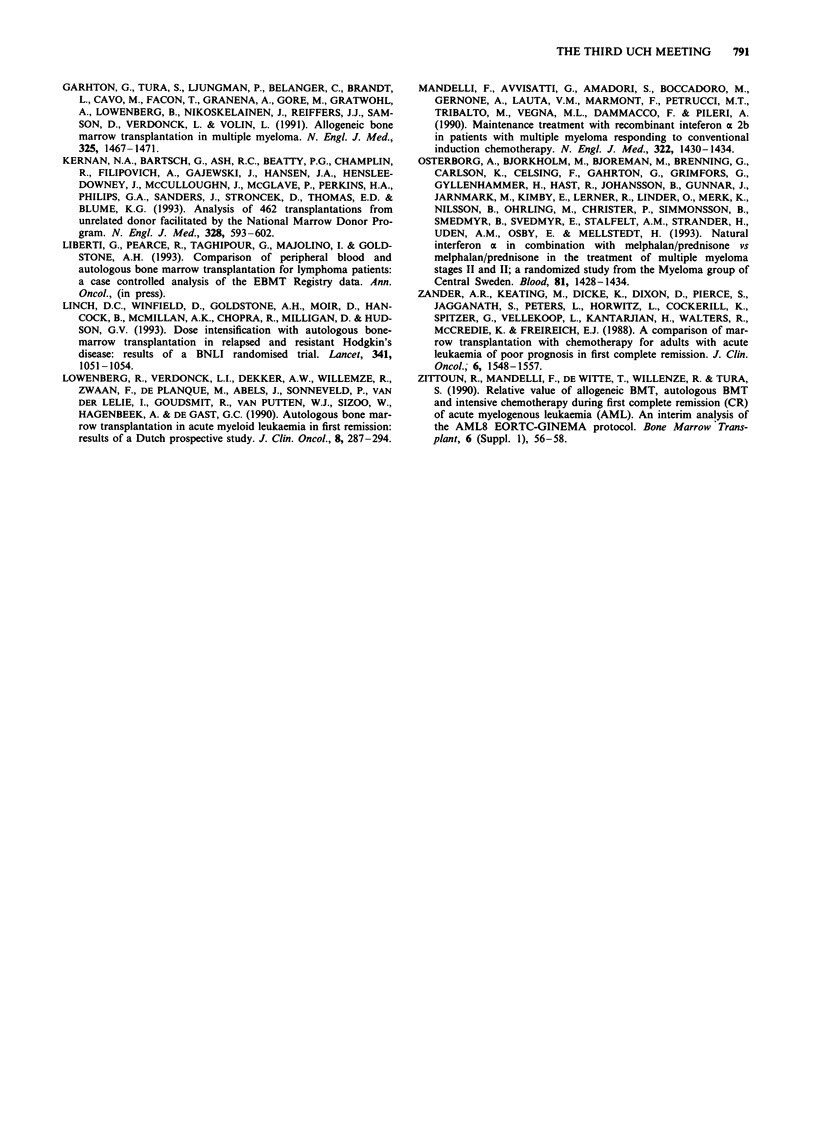# High-dose therapy: the Third UCH Meeting.

**DOI:** 10.1038/bjc.1994.149

**Published:** 1994-04

**Authors:** R. Chopra

**Affiliations:** Department of Haematology, University College London Medical School, UK.


Br. J. Cancer (1994), 69, 788–791 © Macmillan Press Ltd., 1994

MEETING REPORT

### High-dose therapy: the Third UCH Meeting

R. Chopra

*Department of Haematology, University College London Medical School, London, UK.*

The Third UCH High-Dose Meeting was held at University College London in June 1993, in order to review the recent advances and role of high-dose therapy in a variety of malignant disorders. There were 175 participants and a distinguished cast of speakers from Europe and USA.

Historically allogeneic (allo-BMT) and then autologous bone marrow transplantation (ABMT) have been the cornerstone of high-dose therapy, mostly used to treat patients with leukaemia and resistant and relapsed patients with lymphoma. The encouraging results achieved with these treatments have led to their application in other tumours, in particular multiple myeloma, germ cell tumours and breast cancer. Coincident with this increase in the use of high-dose chemotherapy and radiotherapy, there have been advances in biotechnology with the introduction of haemopoietic growth factors and peripheral blood stem cell support. There is now a greater understanding of the histocompatibility system, and the establishment of volunteer bone marrow donor registries has made matched unrelated or volunteer unrelated donor transplants (VUD) an increasingly practical possibility. The aim of the UCH meeting was to review the recent advances and to critically evaluate what impact they make on high-dose therapy. The main themes of the meeting were:

1. the role of high-dose therapy for specific malignancies, in particular breast cancer, multiple myeloma, acute leukaemia and lymphoma;
2. the role of haematopoietic growth factors and peripheral blood stem cell transplantation in high-dose therapy;
3. the role for VUD transplants in childhood leukaemia and chronic myeloid leukaemia (CML) in adults;
4. alternative strategies for the treatment of CML.

### Role of high-dose therapy

Although *in vitro* and animal models have demonstrated a definite and sometimes steep dose–response curve for high-dose chemo/radiotherapy, the clinical evidence for its efficacy is less clear-cut. Initial trials regarding high-dose therapy have involved uncontrolled phase II studies. These studies have enabled drug combinations and toxicity profiles to be identified. They have also allowed the identification of certain categories of patients who may benefit from further dose escalation, but confirmation of these phase II studies through randomised trials is necessary.

### *Acute myeloid leukaemia*

The area in which there is probably the greatest experience to high-dose therapy is in acute leukaemias, and Alan Burnett from Cardiff reviewed its role in acute myeloid leukaemia (AML). The main issue in AML is whether additional high-dose therapy with allogeneic or autologous bone marrow transplantation offers an advantage over consolidation conventional chemotherapy once complete remission is achieved with induction therapy. Single-centre studies and registry data suggest that 40–55% of patients who receive allogeneic or autologous bone marrow transplantation will enjoy prolonged remissions. The results of autologous BMT appear to be equivalent to those of all allogeneic BMT and superior to intensive chemotherapy regimens without transplant. These studies have been criticised because of patient selection biases and 'time censoring'. Approximately 10% of patients in complete remission will relapse within 3 months and therefore will be censored out of transplant trials. In most transplant series the time lapse between achievement of remission and the actual transplant procedure is approximately 3 months, and therefore this 'time censoring' selectively favours the transplant group. Three prospective randomised trials published so far for AML in first remission have assessed the role of allogeneic transplantation. Two have reported a statistically significant advantage for patients receiving allogeneic bone marrow transplantation (Appelbaum *et al*., 1988; Zander *et al*., 1988) and the other study suggested that the approaches are equivalent (Champlin *et al*., 1985). Criticisms of these trials include the assertion that the doses of chemotherapy in the non-transplant arm are not comparable to the newer more intensive consolidation regimens (e.g. high-dose cytosine arabinoside). There are three ongoing studies comparing allo-BMT, ABMT and intensive consolidation chemotherapy. These are the Dutch Hovon study (Lowenberg *et al*., 1990), the EORTC/GIMEMA trial (Zittoun *et al*., 1990) and the UK MRC AML 10 trial. The Dutch study suggests that allogeneic BMT results in fewer relapses than ABMT but that the event-free survival is not statistically different, since the allo-BMT group had a greater procedure-related mortality. The interim analysis of the EORTC/GIMEMA group also shows a greater mortality rate of allo-BMT. The MRC AML 10 trial has accrued 1,400 patients from more than 100 centres in the UK, Ireland and New Zealand. Patients under the age of 55 receive four courses of intensive chemotherapy prior to transplantation. The evaluation of transplantation (allogeneic or autologous) will be its potential additional value compared with no further chemotherapy for the non-transplant group. It is far too early to assess what advantage if any high-dose therapy has, but it was pointed out that approximately 180 patients elected to stop further treatment and were not randomised. This situation has been mirrored in the Dutch and GIMEMA studies, highlighting the fact that even prospective trials can lead to selection biases. Eventually a meta-analysis of all the prospective trials may be required in order to give a clearer indication of the benefits of high-dose therapy.

### *Multiple myeloma*

Diana Sampson from Charing Cross Hospital reviewed the role of high-dose therapy in multiple myeloma. The standard therapy for multiple myeloma has been oral melphalan and prednisone, which produces an objective response in 50–60% of patients and a median survival of 2.5 years. Combination

Correspondence: R. Chopra, Department of Haematology, University College London Medical School, 98 Chenies Mews, London WC1E 6HX, UK.

*Speakers: Alan Burnett, Jacqui Cornish, John Goldman, Diana Sampson, Angello Carella, Vittorio Rizzoli, Phillipe Colombat, Josy Reiffers, Bill Peters, Giovanni Rosti, Jacob Rowe, Jim Armitage, David Linch. Chairman: Tony Goldstone.

Received 7 October 1993; and in revised form 8 November 1993.

chemotherapy such as VBMCP or ABCM, although resulting in an improved initial CR rate, nonetheless gives the same median survival. Moreover, there are no cures. Allo-BMT may result in improved survival but is associated with a high early mortality rate – mostly from infection and graft *vs* host disease (Garhton *et al.*, 1991). Furthermore, allo-BMT in myeloma is only a viable option in those with a suitable donor and younger patients (< 50 years) with a satisfactory performance status. This represents less than 10% of patients with the disease. ABMT is applicable for a larger group of patients, up to 65 years of age, but the long-term survival is similar to conventional chemotherapy. The main problems associated with autologous bone marrow transplantation are an increased relapse rate, marrow contamination and lack of a putative graft *vs* myeloma effect. The role of α-interferon in multiple myeloma for primary therapy still remains to be clarified, with the Italian study (Mandelli *et al.*, 1990) showing a survival advantage for patients receiving α-interferon after VAD therapy and the Scandanavian study (Osterborg *et al.*, 1993) showing no survival advantage. Adjuvant α-interferon appears to prolong the plateau phase after ABMT in the Royal Marsden study, and with a follow-up of 24 months gives a survival advantage of 12 months in the α-interferon arm. Longer follow-up is obviously required for this important group of patients, to ascertain whether adjuvant treatment with α-interferon leads to durable remissions or even 'cure'.

### *Breast cancer*

Breast cancer is a major cause of morbidity and mortality in young women. Although localised early disease may be cured by local treatment such as surgery with or without radiotherapy, there is evidence that breast cancer is a disease in which there is early tumour dissemination. Under these circumstances chemotherapy may be beneficial. Indeed adjuvant chemotherapy together with hormonal manipulation does prolong survival. However, is high-dose therapy of any benefit and when should it be used? Bill Peters presented the experience from Duke University. Initially high-dose chemotherapy (cyclophosphamide, cisplatin and carmustine) was used in poor-prognosis relapsed patients with metastatic disease. There was a high frequency of response, but these responses were not durable, with patients relapsing at sites of previous bulk disease. Subsequently patients received an anthracycline-based regimen, AFM (doxorubicin, 5-fluorouracil and methotrexate), to achieve cytoreduction. Those with a complete remission and partial response proceeded to high-dose chemotherapy and ABMT. There was an 18% toxic death rate with significant pulmonary toxicity. However 20% of these poor-prognosis patients are alive and disease free at 5 years. Although these data are highly promising and suggest that dose escalation may be beneficial in breast cancer, it was emphasised that randomised trials are needed. The Cancer and Leukemia Group B (CALGB) are carrying out such trials in two situations: for relapsed patients with stage 4 metastatic disease and for patients with primary disease with ten or more involved axillary lymph nodes.

### The role of haemopoietic growth factors and peripheral blood stem cell transplantation in high-dose therapy

Peripheral blood stem cells (PBSCs) to abrogate myelosuppression after high-dose chemotherapy are now being used increasingly, and the state of the art was summarised by Josy Reiffers (Bordeaux). He reviewed the biology of peripheral blood stem cells, highlighting the fact that peripheral blood contains pluripotent stem cells [CD34 + ve; rhodamine dull; quiescent (resistant to 5-FU and cytosine arabinoside); respond to combinations of cytokines such as IL-1, IL-3 and stem cell factor; and possess self-renewal capacity]. The main advantage of the use of peripheral stem cells in transplantation is the rapid return of both neutrophil and platelet populations after high-dose chemotherapy. This is only seen when the patient receives priming with either chemotherapy, haemopoietic growth factors or a combination of the two. The optimal priming has yet to be evaluated. The data suggest that a combination of granulocyte–macrophage colony-stimulating factors (GM-CSF) and chemotherapy such as high-dose cyclophosphamide ($7\ g\ m^{-2}$) yields the most peripheral blood progenitors. Peripheral stem cells could permit engraftment after most high-dose regimens including total body irradiation and busulphan/cyclophosphamide, but whether long-term engraftment will be sustained over a period of years remains to be seen. Patients who have received peripheral blood stem transplants for acute leukaemia had a delayed platelet recovery, whereas those with lymphoma and multiple myeloma had rapid engraftment.

Other potential advantages for PBSC transplants include fewer infections and a reduced procedure-related mortality. Initial data suggest that infections and antibiotic usage may be decreased, but the impact on procedure-related mortality remains unproven. Although most patients have a shorter duration of hospital stay due to neutropenia, the cost benefits of chemotherapy and haemopoietic growth factor-primed PBSC transplants should be evaluated by taking into account time spent in hospital for the chemotherapy and haemopoietic growth factor administration, and the resources required for stem cell collections, particularly if multiple aphereses are performed. David Linch (London) presented the Univerity College Hospital experience of the use of $1.5\ g\ m^{-2}$ cyclophosphamide and $5-10\ \mu g\ kg^{-1}$ G-CSF used to prime 18 patients with relapsed and resistant malignant lymphomas. The patients then received BEAM (BCNU, etoposide, cytosine arabinoside and melphalan) chemotherapy and could be rescued with PBSCS from a single apheresis. The priming chemotherapy and haemopoietic growth factor were administered on an outpatient basis without any associated morbidity. The optimal time for PBSC collection was consistently at day 9 or 10 after the cyclophosphamide. Marrow recovery for both neutrophils and platelets was shortened by 7 and 11 days respectively as compared with BEAM and ABMT. G-CSF administered after PBSC infusion did not have a major impact on engraftment.

A major issue is whether PBSCs are preferable to bone marrow-derived stem cells from the perspective of disease eradication. There is no clear evidence that PBSC transplants have a lower disease contamination, the progression-free survival of patients reported to the EBMT lymphoma registry receiving peripheral blood stem cells being no different from those receiving autologous marrow (Liberti *et al.*, 1993). Jim Armitage (Omaha), however, presented an interesting albeit retrospective analysis of the data from Nebraska for patients with NHL who had undergone high-dose therapy with either ABMT or a PBSC transplant. The patients in the PBSC group had a significantly improved progression-free survival even though they had poorer prognostic features. This result requires confirmation in a randomised trial.

The use of PBSCs may permit the safer use of high-dose therapy, at least from the point of view of haematological toxicity. However, what is the precise role of high-dose therapy? In Hodgkin's disease there is some evidence that high-dose therapy may lead to long-term survival in resistant and refractory patients. This has been confirmed in a small randomised trial (Linch *et al.*, 1993). There are nonetheless a group of poor-prognosis relapsed and refractory patients who may benefit from further dose escalation. However, this will invariably lead to non-haematological toxicity: e.g. high-dose BCNU causing pneumonitis, and etoposide causing mucositis and GI haemorrhage. This will not be ameliorated by existing growth factors or PBSC transplants. In non-Hodgkin's lymphoma, the role of high-dose chemotherapy is generally accepted for patients with relapsed but chemosensitive disease, although confirmation is awaited from ongoing randomised trials. Indeed, in the lymphoma field, despite the large number of high-dose procedures carried out, there have been few randomised trials supporting this therapy. This is in marked contrast to the situation for breast cancer in the

USA, where large numbers of patients have been randomised between 'conventional therapy' and high-dose therapy with ABMT/PBSCs.

**Volunteer unrelated donor transplantation**

Jacqui Cornish from Bristol Children's Hospital presented preliminary data on their experience of volunteer unrelated donor (VUD) transplants. Forty-seven children (age 3 months to 17 years) received either a fully matched (28 patients) or a mismatched marrow (19 patients), the majority with relapsed acute leukaemia. Graft *vs* host disease (GVHD) prophylaxis consisted of T-cell depletion and cyclosporin A. Four patients did not engraft. The prevalence of severe graft *vs* host disease was 16% for the fully matched group and 23% for the mismatched group. The main cause of death was not procedure related but relapse. There was no statistically significant difference in relapse rate between the matched and the mismatched group. The follow-up in these patients is too short to make a meaningful assessment of long-term outcome. It would appear that in children the incidence of graft *vs* host disease is lower than in adults, as is the procedure-related toxicity. Furthermore, one locus HLA mismatch may not be a contraindication to its use as donor marrow in children. In contrast, John Goldman (London) highlighted the problems of VUD transplants in adults with chronic myeloid leukaemia, for which there is now an increasing number of data (Kernan *et al.*, 1993). Unrelated donor transplants are associated with a higher incidence of engraftment problems, an increased incidence of acute and chronic GVHD, an increase in infections and poorer overall survival then sibling donor transplants. Furthermore, there appears to be no difference in the relapse rate, suggesting that there is as yet no proven additional graft *vs* leukaemia effect for unrelated donor transplants. The increased incidence of GVHD in matched unrelated patients is further evidence that conventional serology for class I and class II HLA antigens, together with mixed lymphocyte cultures (MLCs), is not sensitive enough in detecting donor/recipient disparities. Newer methods such as analysis of restriction length polymorphisms, allospecific oligonucleotide typing, cytotoxic T-cell precursor and helper T-cell precursor assays may be better at predicting GVHD, thus enabling a clearer assessment of outcome. VUD transplants therefore remain an experimental approach and in CML, at least, alternatives such as autologous bone marrow transplantation should be explored.

**Alternative therapies for chronic myeloid leukaemia**

Alternative therapies for CGL were summarised by John Goldman and these include the use of α-interferon and high-dose hydroxyurea and allogeneic, volunteer donor unrelated and autologous transplants.

The use of autologous bone marrow transplantation is based on the premise that normal (Philadelphia chromosome negative; bcr/abl negative) early progenitors or stem cells persist in the marrow of CML patients. There have been attempts to select out these normal progenitors. These include increasing Ph-negative cells in long-term bone marrow cultures, using the loss of stromal adhesion properties in CML to select adherent 'normal' cells, and the use of $CD34^+$ $DR^-$ or CD $34^+$/Epo receptor$^+$ cells to restore normal haemopoiesis. These *in vitro* manipulations are by their very nature painstaking, and an alternative *in vivo* approach to selecting Ph-negative cells was presented by Angelo Carella (Genoa). The Genoa group have selected 66 patients with CML (refractory to 6 months of initial α-interferon therapy and under the age of 60 years) for treatment with myelo-ablative idarubicin, cytosine arabinoside and etoposide. On early recovery from the aplastic phase patients underwent leucapheresis, the premise being that a greater proportion of the progenitors at this stage will be Ph negative. Ten of these patients received total body irradiation and were rescued with the peripheral stem cells. There was prolonged marrow recovery and median time to $0.1 \times 10^9 l^{-1}$ neutrophils and $50 \times 10^9 l^{-1}$ platelets was 18 and 54 days respectively. Two patients died from aplasia. However, five out of eight evaluable patients are Ph negative, which is undoubtedly an impressive result, although conceptually difficult to reconcile with the belief that in CML there is a premature release of Ph-positive progenitor cells from the bone marrow secondary to the abnormal loss of adhesion to the stroma (Dunbar & Stewart, 1992).

**Future trends**

The meeting highlighted the fact that the use of high-dose therapy is evolving rapidly. Conventional consolidation therapies that do not require stem cell support are being introduced. The choice of techniques for stem cell support is increasing. Initially only allogeneic and subsequently autologous bone marrow-derived stem cells were used. Increasingly peripheral blood stem cells (both autologous and allogeneic) will be used. There is no doubt that these allow more rapid engraftment than marrow-derived progenitors, but in the case of allogeneic peripheral stem cells there may be a greater risk of GVHD since T cells will also be mobilised. Both bone marrow-derived and peripheral blood stem cells have now been used to purify $CD34^+$ progenitor cells using monoclonal antibodies. This has the theoretical advantage of reducing contamination with tumour cells and would be of potential benefit when there is overt bone marrow involvement with disease. The monoclonal antibodies may be biotinylated as in the CellPro system (Berenson *et al.*, 1991). Marrow is then incubated with the antibody and passed down a column of beads which are coated with avidin. The biotin binds to the avidin, resulting in the progenitor cells remaining behind in the column. After the remaining marrow cells have passed through the column the progenitors are removed by agitation. Although initial results with this technique are encouraging from the point of view of engraftment, whether it makes an impact on disease-free survival remains to be proven.

*Ex vivo* expansion of PBSCs using a cocktail of haemopoietic growth factors has been advocated in situations in which limited numbers of stem cells are available, for example because of heavy pretreatment with alkylating agents. The problem with this approach is that *ex vivo* expansion also results in cell differentiation, so that although there may be initial engraftment it may not be sustained as a result of the loss of primitive stem cells.

The technology required to deliver high-dose therapy safely is now being optimised; the central issue as to whether high-dose therapy cures more patients will need to be answered in some of the large-scale trials that are under way and will be reported at the next UCH high-dose meeting.

**References**

APPELBAUM, F.R., FISHER, L.D., THOMAS, E.D. & THE SEATTLE MARROW TRANSPLANT TEAM (1988). Chemotherapy *vs* marrow transplanation for adults with acute non-lymphoblastic leukaemia; a five year follow up. *Blood*, **72**, 179-184.

BERENSON, R., BENSINGER, W., HILL, R., ANDREWS, R.G., GARCIA-LOPEZ, J., KALAMASZ, D.F., STILL, B.J., SPITZER, G., BUCKNER, C.D., BERNSTEIN, I.D. & THOMAS, E.D. (1991). Engraftment after infusion of $CD34^+$ marrow cells in patients with breast cancer or neuroblastoma. *Blood*, **77**, 1717-1722.

CHAMPLIN, R.E., HO, W.G., GALE, R.P., WINSTON, D., SELCH, M., MISUYASU, R., LENARSKY, C., ELASHOFF, R., ZIGHELBOIM, J., FEIG, S.A. (1985). Treatment of acute myelogenous leukaemia: a prospective controlled trial of bone marrow transplantation *vs* consolidation chemotherapy. *Ann. Int. Med.*, **10**, 285-291.

DUNBAR, C.E. & STEWART, F.M. (1992). Separating the wheat from the chaff: selection of benign hematopoietic cells in chronic myeloid leukemia. *Blood*, **79**, 1107-1110.

GARHTON, G., TURA, S., LJUNGMAN, P., BELANGER, C., BRANDT, L., CAVO, M., FACON, T., GRANENA, A., GORE, M., GRATWOHL, A., LOWENBERG, B., NIKOSKELAINEN, J., REIFFERS, J.J., SAMSON, D., VERDONCK, L. & VOLIN, L. (1991). Allogeneic bone marrow transplantation in multiple myeloma. *N. Engl. J. Med.*, **325**, 1467–1471.

KERNAN, N.A., BARTSCH, G., ASH, R.C., BEATTY, P.G., CHAMPLIN, R., FILIPOVICH, A., GAJEWSKI, J., HANSEN, J.A., HENSLEE-DOWNEY, J., MCCULLOUGHN, J., MCGLAVE, P., PERKINS, H.A., PHILIPS, G.A., SANDERS, J., STRONCEK, D., THOMAS, E.D. & BLUME, K.G. (1993). Analysis of 462 transplantations from unrelated donor facilitated by the National Marrow Donor Program. *N. Engl. J. Med.*, **328**, 593–602.

LIBERTI, G., PEARCE, R., TAGHIPOUR, G., MAJOLINO, I. & GOLDSTONE, A.H. (1993). Comparison of peripheral blood and autologous bone marrow transplantation for lymphoma patients: a case controlled analysis of the EBMT Registry data. *Ann. Oncol.*, (in press).

LINCH, D.C., WINFIELD, D., GOLDSTONE, A.H., MOIR, D., HANCOCK, B., MCMILLAN, A.K., CHOPRA, R., MILLIGAN, D. & HUDSON, G.V. (1993). Dose intensification with autologous bone-marrow transplantation in relapsed and resistant Hodgkin's disease: results of a BNLI randomised trial. *Lancet*, **341**, 1051–1054.

LOWENBERG, R., VERDONCK, L.I., DEKKER, A.W., WILLEMZE, R., ZWAAN, F., DE PLANQUE, M., ABELS, J., SONNEVELD, P., VAN DER LELIE, I., GOUDSMIT, R., VAN PUTTEN, W.J., SIZOO, W., HAGENBEEK, A. & DE GAST, G.C. (1990). Autologous bone marrow transplantation in acute myeloid leukaemia in first remission: results of a Dutch prospective study. *J. Clin. Oncol.*, **8**, 287–294.

MANDELLI, F., AVVISATTI, G., AMADORI, S., BOCCADORO, M., GERNONE, A., LAUTA, V.M., MARMONT, F., PETRUCCI, M.T., TRIBALTO, M., VEGNA, M.L., DAMMACCO, F. & PILERI, A. (1990). Maintenance treatment with recombinant inteferon α 2b in patients with multiple myeloma responding to conventional induction chemotherapy. *N. Engl. J. Med.*, **322**, 1430–1434.

OSTERBORG, A., BJORKHOLM, M., BJOREMAN, M., BRENNING, G., CARLSON, K., CELSING, F., GAHRTON, G., GRIMFORS, G., GYLLENHAMMER, H., HAST, R., JOHANSSON, B., GUNNAR, J., JARNMARK, M., KIMBY, E., LERNER, R., LINDER, O., MERK, K., NILSSON, B., OHRLING, M., CHRISTER, P., SIMMONSSON, B., SMEDMYR, B., SVEDMYR, E., STALFELT, A.M., STRANDER, H., UDEN, A.M., OSBY, E. & MELLSTEDT, H. (1993). Natural interferon α in combination with melphalan/prednisone *vs* melphalan/prednisone in the treatment of multiple myeloma stages II and II; a randomized study from the Myeloma group of Central Sweden. *Blood*, **81**, 1428–1434.

ZANDER, A.R., KEATING, M., DICKE, K., DIXON, D., PIERCE, S., JAGGANATH, S., PETERS, L., HORWITZ, L., COCKERILL, K., SPITZER, G., VELLEKOOP, L., KANTARJIAN, H., WALTERS, R., MCCREDIE, K. & FREIREICH, E.J. (1988). A comparison of marrow transplantation with chemotherapy for adults with acute leukaemia of poor prognosis in first complete remission. *J. Clin. Oncol.*; **6**, 1548–1557.

ZITTOUN, R., MANDELLI, F., DE WITTE, T., WILLENZE, R. & TURA, S. (1990). Relative value of allogeneic BMT, autologous BMT and intensive chemotherapy during first complete remission (CR) of acute myelogenous leukaemia (AML). An interim analysis of the AML8 EORTC-GINEMA protocol. *Bone Marrow Transplant*, **6** (Suppl. 1), 56–58.